# Microheterogeneity of transthyretin in serum and ascitic fluid of ovarian cancer patients

**DOI:** 10.1186/1471-2407-5-133

**Published:** 2005-10-17

**Authors:** Beate Gericke, Jens Raila, Jalid Sehouli, Sophie Haebel, Dominique Könsgen, Alexander Mustea, Florian J Schweigert

**Affiliations:** 1 Department of Physiology and Pathophysiology, Institute of Nutritional Science, University of Potsdam, Arthur-Scheunert-Allee 114-116; D-14558 Nuthetal, Germany; 2 Department of Obstetrics and Gynecology, Campus Virchow-Klinikum, Charité Universitätsmedizin Berlin, Augustenburger Platz 1, 13353 Berlin, Germany; 3 Interdisciplinary Center for Mass Spectrometry of Biopolymers, University of Potsdam, Karl-Liebknecht-Str. 24-25, D-14476 Golm, Germany

## Abstract

**Background:**

Transthyretin (TTR), a traditional biomarker for nutritional and inflammatory status exists in different molecular variants of yet unknown importance. A truncated form of TTR has recently been described to be part of a set of biomarkers for the diagnosis of ovarian cancer. The main aim of the study was therefore to characterize differences in microheterogeneity between ascitic fluid and plasma of women affected with ovarian cancer and to evaluate the tumor site as the possible source of TTR.

**Methods:**

Subjects were 48 women with primary invasive epithelial ovarian cancer or recurrent ovarian carcinoma. The control group consisted of 20 postmenopausal women. TTR and retinol-binding protein (RBP) levels were measured by enzyme-linked immunoassay (ELISA) and C-reactive protein (CRP) levels by a high-sensitivity latex particle turbidimetric assay. The molecular heterogeneity of TTR was analysed using immunoprecipitation and matrix-associated laser desorption ionization time-of-flight mass spectrometry (MALDI-TOF-MS). Presence of TTR in tumor tissue was determined with indirect peroxidase immunostaining.

**Results:**

TTR and RBP (μg/ml) levels in serum were 148.5 ± 96.7 and 22.5 ± 14.8 in affected women compared to 363.3 ± 105.5 and 55.8 ± 9.3 in healthy postmenopausal women (p < 0.01). In ascitic fluid, levels were 1.02 ± 0.24 and 4.63 ± 1.57 μg/ml, respectively. The mean levels of TTR and RBP in serum showed a tendency to decrease with the severity of the disease and were lower in affected women whose CRP levels were > 40 mg/ml (p = 0.08 for TTR; p < 0.05 for RBP). No differences in TTR microheterogeneity were observed between TTR isolated from serum of affected and healthy women or from ascitic fluid. TTR occurred rather consistently in four variants. Mass signals were at 13758 ± 7, 13876 ± 13 (greatest intensity), 13924 ± 21 and 14062 ± 24 Da, representing native, S-cysteinylated, S-cysteinglycinylated and glutathionylated TTR, respectively. Serum of healthy and affected women as well as ascitic fluid contained the truncated fragment of TTR (12828 ± 11 Da). No immunoreactive TTR was observed in the tumor sites.

**Conclusion:**

The severity of the cancer associated catabolism as well as the inflammation status affect serum TTR and RBP levels. Neither TTR nor its truncated form originates from tumor tissue and its occurrence in ascites may well reflect the filtration from blood into ascitic fluid.

## Background

TTR, formerly called prealbumin, belongs to a group of proteins including thyroxine-binding globulin and albumin which bind to and transport thyroid hormones in the blood. TTR is also involved in the metabolism of vitamin A as it binds RBP, the specific plasma transport protein for retinol. First identified in 1942 by Kabat et al. [1] in serum and cerebrospinal fluid, TTR has been described as a so-called visceral protein that is synthesized in the liver in response to nutritional supply. TTR plasma levels can thus be used as a sensitive biochemical parameter of subclinical malnutrition, because both the synthesis of proteins as well as energy intake are reflected in its serum levels. Serum levels of TTR, however, are also affected by acute and chronic diseases associated with an acute-phase response. Under these conditions, liver activity is converted to the synthesis of acute-phase response proteins, resulting in a drop in visceral proteins despite adequate nutritional supply [2-5].

Epithelial ovarian cancer is the leading cause of death from gynaecologic malignancies in western countries [6]. Most patients are first diagnosed at an advanced stage with diffuse peritoneal metastasis outside the pelvis (FIGO stage III or IV). Tumor stage is one of the most important prognostic factors in ovarian cancer [7]. The 5-year survival rate for FIGO stage III ranges from 25 to 45%, whilst for patients diagnosed at FIGO stage I or II survival rates are between 85 and 95% [8]. Therefore, various strategies have been investigated to increase the detection rate of early ovarian cancer. A systematic review by Fung and co-workers [9] on the screening of postmenopausal women for ovarian cancer showed that for every 10,000 women participating in an annual screening program with cancer associated antigen 125 (CA125) over 3 years, 800 have had an ultrasound scan due to an elevated CA125, 30 underwent surgery because of an abnormal ultrasound, whilst only 6 women were diagnosed with ovarian cancer of whom only three where detected at an early stage. Therefore, despite the combination of CA125 monitoring and ultrasound this strategy remains insufficient as a screening tool.

Unfortunately, most other biomarkers also have low sensitivity and specificity and little predictive value [10]. Application of new technologies for detection of ovarian cancer could have an important effect on public health [11], but to achieve this goal, specific and sensitive molecular markers are essential [12,13]. This need is especially urgent in women, who have a high risk of ovarian cancer due to family or personal history of cancer, and for women with a genetic predisposition to cancer due to abnormalities in predisposition genes [14]. Besides protein profiling, the determination of changes in the microheterogeneity of a variety of proteins has been suggested as an approach to biomarker discovery [15].

In plasma, the non-glycosylated TTR is present as a tetramer of non-covalently bound monomers of ~14 kDa. Physiologically its importance is related to the transport of thyroid hormones and retinol. The association of TTR and RBP is a prerequisite for the homeostatic control of plasma and retinol levels. In addition to mutations on protein level, TTR exists in different isoforms [16,17]. The isoforms results when the cyteine residue on position 10 (Cys^10^) makes a mixed disulfide with the amino acid cysteine the peptide cysteinyl-glycine, or the peptide glutathione. The possible importance of this as a risk factor for the onset of senile systemic amyloidosis remains to be elucidated [18,19]. Additionally, Cys^10 ^adducts of S-homocysteine of TTR have been detected in plasma of humans with hyper-homocysteinemia [20]. Recently, a new truncated variant of TTR together with apolipoprotein A1 and a cleaved fragment of inter-α-trypsin inhibitor heavy chain H4 were described as an efficient set of new biomarkers for ovarian cancer in women [21].

In human primary hepatic cancer, the mRNA of TTR, which is normally highly expressed in the liver, is strikingly suppressed [22]. Not only in hepatic cancer, but also for broncho-pulmonary carcinoid cancers, TTR was concluded to be a useful marker [23]. Nothing however is known with regard to ovarian cancer as a source of TTR itself or cleavage products thereof. Since we have recently shown that TTR is present in ascitic fluid [24], it cannot be excluded that its presence might not only arise from an increased permeability for blood constituents into ascitic fluid due to an intensified vascularization [25], but may also reflect the secretion of products synthesized by the malignant ovary cells due to the intimate contact between tumor site and ascitic fluid.

The study was thus conducted to primarily characterize possible differences in microheterogeneity of TTR arising from posttranslational modifications and/or products of protein degradation or proteolysis between serum and ascitic fluid of women with ovarian cancer and also to evaluate the affected ovary as a possible site of TTR expression.

## Methods

### Subjects

The study was conducted on 48 patients (average age 53 ± 11.9; mean ± SD) with ovarian cancer admitted to the Department of Gynecology and Obstetrics, Charité, Campus Virchow-Klinikum, Berlin, Germany. All patients underwent primary surgery with median laparotomy, hysterectomy, adenectomy, omentectomy and pelvic and paraortal lymphadenectomy to achieve maximal tumor reduction. According to the classifications of the International Federation of Gynecology and Obstetrics (FIGO), the treated group consisted of four patients with stage Ic, two with stage IIc, 28 with stage III and 14 with stage IV. Of the 48 women, 25 were suffering from primary ovarian carcinoma and 23 had recurrent ovarian carcinoma. The controls were 20 healthy postmenopausal women (average age 58 ± 1.0; mean ± SD). The postmenopausal status was proved by the assessment of LH and FSH. The study protocol was approved by the hospitals and University of Potsdam Ethics Committee. All samples and relevant clinical data were obtained from the Tumor Bank Ovarian Cancer (TOC). Informed consent was obtained from each participant.

Ascitic fluid was collected under sterile conditions from the patients with ovarian carcinoma and centrifuged at 1500 × *g *for 20 min at 4°C. The supernatants were stored at -80°C. Serum was separated from freshly drawn blood at the same time of paracentesis and stored at -80°C until assayed.

Tissue samples were fixed in 4% PBS-buffered paraformaldehyde for 24 h and embedded in paraplast. Each of the samples was stained routinely with haematoxylin and eosin (H+E) and diagnosed. All tumor samples were reviewed by a pathologist.

### Determination of TTR, RBP and CRP levels

Levels of TTR and RBP in serum and ascitic fluid were determined by ELISA using polyclonal rabbit anti-human antibodies (DakoCytomation, Hamburg, Germany) [17]. CRP levels in serum were measured with a high sensitivity latex turbimetric immunoassay using a latex-coupled monoclonal mouse anti-human antibody (Olympus AU 600, Biomed, Germany). The sensitivity of this assay was 0.005 mg/dl. The 90^th ^percentile of normal CRP distribution was 0.3 mg/dl.

### Immunoprecipitation of TTR and subsequent analysis by MALDI-TOF-MS

TTR from serum and ascitic fluid of 20 randomized representative women was prepared by immunoprecipitation. The subgroup consisted of two patients with FIGO stage Ic or IIc, 13 with stage III and 5 with stage IV. Briefly, 15 μl of serum or ascitic fluid was treated with an equal amount of a polyclonal rabbit anti human antibody (DakoCytomation). The mixture was incubated for two hours at 37°C and then centrifuged at 15.000 × g for 15 min at room temperature. The supernatant was removed and the immunoprecipitated complex of TTR and antibody was then washed with high performance liquid chromatography grade water.

To determine the disulfide linkage of TTR adducts, the immunoprecipitated TTR was treated with dithiothreitol (DTT). DTT solution, 100 mM in buffer (100 mM NH_4_CO_3_, pH 8.8) was added to the solution at a ratio of 1:1 (DTT solution volume/TTR solution volume). The mixture was incubated for 2 h at room temperature and precipitated samples were subsequently subjected to MALDI-TOF-MS.

MALDI mass spectra of the precipitated TTR from serum and ascitic fluid were obtained using a Reflex II MALDI-TOF mass spectrometer (Bruker-Daltonik, Bremen, Germany). MALDI-TOF MS of serum samples was performed in linear mode at 20 k acceleration voltage using sinapic acid as matrix. For ionization, a nitrogen laser (337 nm, 3 ns pulse width, 3 Hz) was used. The samples were prepared in a two step procedure: First, 0.5 μl serum were deposited on the target. Secondly, 0.5 μl saturated sinapinic acid solution was placed on serum drop and dried. This step was repeated. The matrix solution contained 1 mg sinapinic acid and equal amounts (25 μl) 1% trifluoroacetic acid and acetonitrile. For optimisation of the mass spectra, the laser was aimed either at the central area of the sample or at the outmost edge of the crystal rim. All spectra were measured using external calibration.

### Immunohistochemistry of TTR

For indirect peroxidase immunostaining of TTR, slides were deparaffinized, rehydrated in a decreased series of alcohol to water and exposed for 60 min in 0.5% hydrogen peroxide in methanol in order to deactivate endogenous peroxidases. Non-specific antibody binding was blocked for 30 min in Tris-buffered saline (TBS, pH 7.6) containing 5% bovine serum albumin (BSA; Sigma, Taufkirchen, Germany). The primary human anti-TTR antibody (DakoCytomation) was diluted 1:100 in 1% bovine serum albumin (BSA) in TBS. After overnight incubations at 4°C, the sections were treated with peroxidase-coupled swine anti-rabbit IgG (DakoCytomation) diluted 1:100 in 1% BSA in TBS for 30 min. The antigen-antibody binding sites were visualized by incubating the sections in a solution of diaminobenzidine tetrahydrochloride (DAB; Sigma) containing 0.01% hydrogen peroxide in 0.1 M imidazole buffer (pH 7.1). Counterstaining was performed with Papanicolaou hematoxylin. Negative controls, which included the omission of the primary antibodies, revealed no significant labelling. A positive control (liver) was included in each individual staining process. The sections were examined and photographed with an Olympus BX-50 microscope equipped with a ColorView 12 CCD video camera (SIS, Münster, Germany). Images were processed using analySIS™ 3.0 software (SIS).

### Statistical procedures

Values are expressed as means and standard deviations (SD). Unpaired *t *tests were performed to compare serum values with ascitic fluid or to compare between the groups using standard methods software (SPSS package, version 10.0). P < 0.05 was regarded as statistically significant.

## Results

### TTR and RBP levels in serum and ascitic fluid

**Figure 1:**
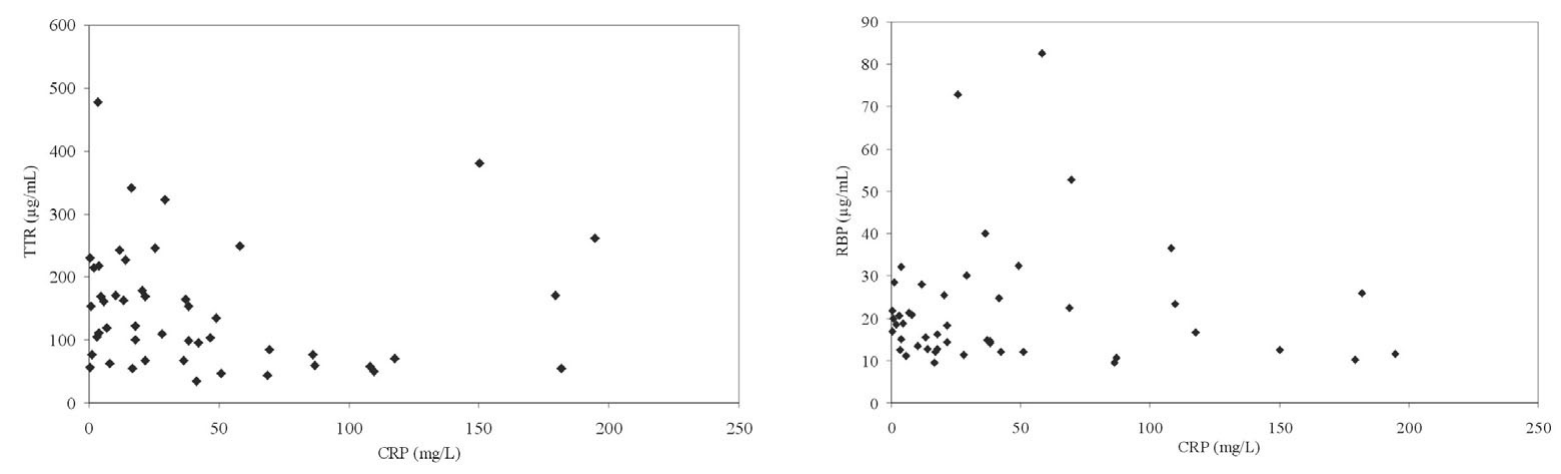
Correlation between levels of CRP and TTR in serum (p = 0.08) and between levels of CRP and RBP in ascitic fluid (p < 0.05).

Results of serum and ascitic fluid levels of TTR and RBP are shown in Table 1. In women with cancer, serum levels of both TTR and RBP were lower compared to healthy controls (p < 0.01). In the more severe stages of ovarian cancer the levels showed a tendency to be even lower. Within the cancer group, increased levels of CRP (cut-off > 40 mg/ml) in serum were associated with lower levels of TTR (p = 0.08) and RBP (p < 0.05) (Fig. 1). TTR and RBP levels in ascitic fluid were substantially lower compared to serum (p < 0.01). No obvious differences of TTR and RBP concentration in ascitic fluid between FIGO stages were observed.

### TTR microheterogeneity in serum and ascitic fluid

**Figure 2:**
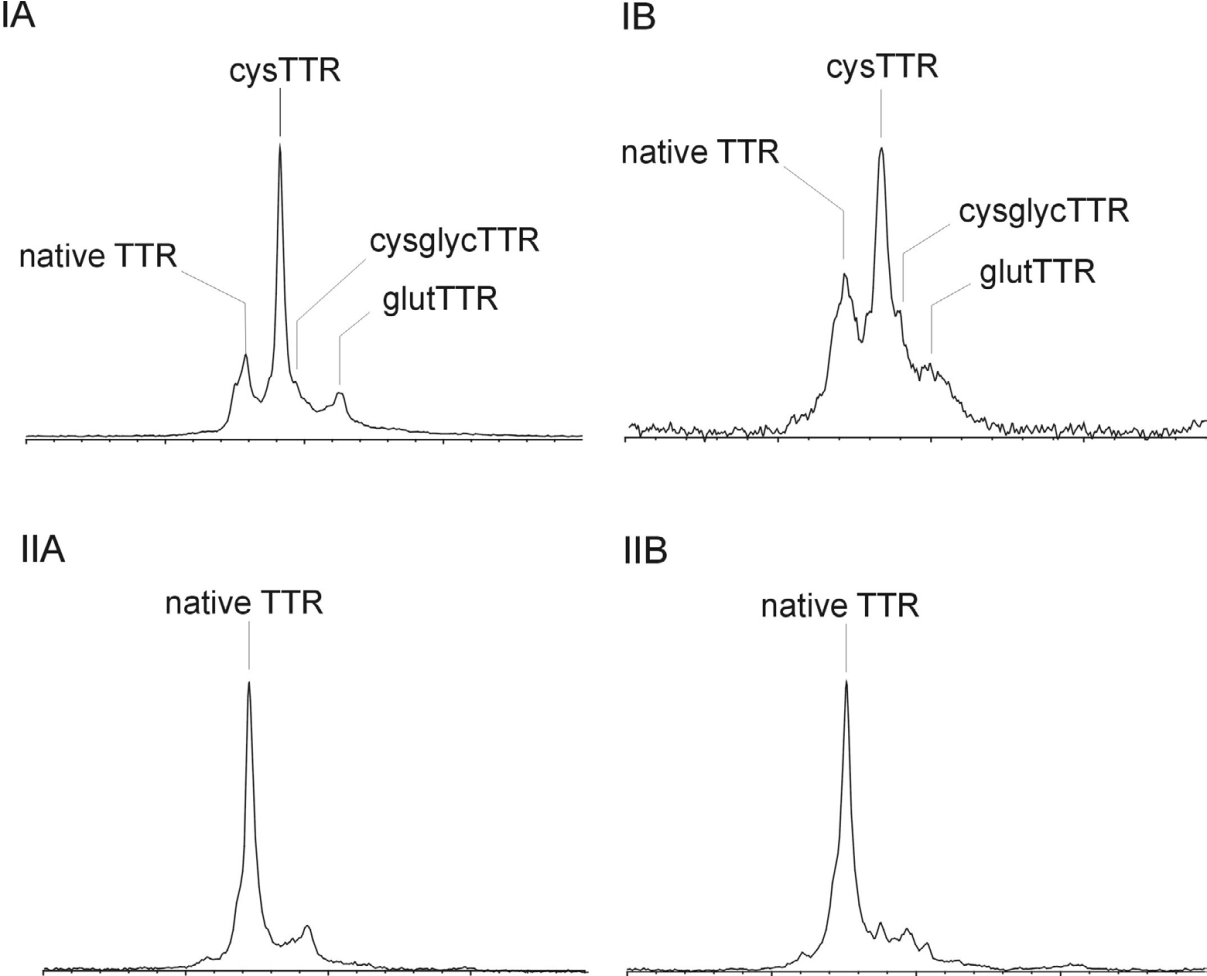
Mass spectra resulting from MALDI-TOF-MS after immunoprecipitation of TTR in plasma (A) and ascites (B) obtained from women with ovarian cancer. The untreated TTR (I) and the TTR after treatment with dithioretiol (II) are shown.

Using the combination of immunoprecipitation and subsequent MALDI-TOF-MS we were able to show that no obvious differences exists in the microheterogeneity of TTR between serum of affected and healthy women as well as in ascitic fluid. TTR monomer occurred rather consistently in four major variants in the range where TTR and its conjugated forms should normally appear (m/z 13,700 – 14,100). The results are summarized in Table 2. In the mass spectra of serum and ascitic fluid (Fig. 2 and Tab. 2) peaks dominated at m/z 13,875.8 ± 12.8 and 13,876.9 ± 13.3 respectively. Three additional mass spectra were recorded. The mass differences between these variants were similar in serum and ascitic fluid (Tab. 2). The molecular mass of 13,757.7 ± 7.1 Da corresponded to the native, unmodified TTR. The other peaks in serum representing Cys^10 ^adducts for S-cysteine (TTR- Cys^10^-S-S-Cys, mass = 13,875.8 ± 12.8 Da), S-cysteinylglycine (TTR- Cys^10^-S-S-CysGly, mass = 13,923.6 ± 21.0) and S-glutathione (TTR- Cys^10^-S-S-SG, mass = 14,062.1 ± 24.7). The shift in the mass spectrum of TTR after treatment with DTT, towards the native form of TTR, indicates that the adducts are formed via the disulfide linkage at Cys^10 ^(Fig. 2). Additionally, in serum and ascitic fluid a smaller mass signal could be observed with varying intensity at a molecular mass of 12828 ± 11 Da.

### Immunohistochemistry of TTR

**Figure 3:**
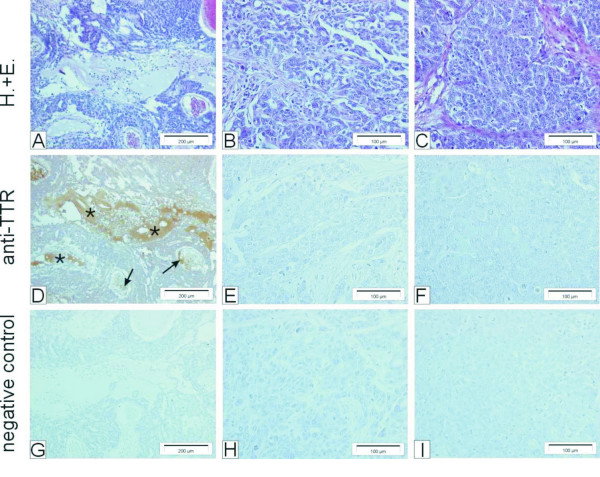
Histological sections from ovarian malignoma subjected to staining with H+E (A-C) or immunodetection of TTR (D-E). Diffuse TTR immunostaining was only detectable within blood vessels (arrows) or plasma insudations (asterisks) (D). No immunoreactivity was observed within epithelial cells of any tumour specimen (D-F)). Negative controls, which included the omission of the primary antibody, revealed no significant labelling (G-I).

In order to assess the expression of TTR within ovarian cancer tissue we performed immunohistochemical staining using a polyclonal TTR antibody in paraffin embedded sections. TTR immunoreactivity was previously tested in human liver sections and revealed cytoplasmic staining within hepatocytes (data not shown). In ovarian cancer tissues diffuse TTR immunostaining was only observed within blood vessels, haemorrhages or plasma insudations (Fig. 3). No TTR labelling however was seen within the epithelial cells of any cancer specimen.

## Discussion

Epithelial ovarian cancer is the leading cause of death from gynaecologic malignancies in western countries [26,27]. The tumor stage at time of diagnosis and the postoperative residual tumor mass are important prognostic factors and are unequivocally related to overall survival [26]. Other prognostic factors are identified mostly in small series and are the source of controversial discussion in the relevant literature.

Serum TTR is traditionally a valid marker for nutritional status in general and in cancer patients it has gained considerable interest with regard to the use as an early diagnostic marker in ovarian cancer [21]. As depletion of nutritional reserves and a subsequent significant weight loss can lead to an increased risk of morbidity, reduced chemotherapy response, and shorter survival in patients with cancer, TTR is a valid prognostic marker [28]. Interestingly however, TTR and RBP levels of serum are affected not only by the nutritional status of the individual but are also reduced during the acute phase response associated with inflammation [29]. Additionally to quantitative aspects, the TTR molecule in serum exists in numerous variants due either to genetic differences or because of modification on one readily accessible cystein within the molecule. The microheterogeneity is affected by different metabolic aspects such as oxidative stress or homocystein levels [20,30]. Nothing is known however with regard to possible variation due to metabolic alterations in cancer.

The results of the present study confirm previous results for cancer patients in general and especially for patients with ovarian cancer, regarding the greatly reduced serum levels of TTR and RBP [31]. Interestingly however, the intensity of the disease has no significant influence on serum levels, indicating that it is a general phenomenon possibly associated with cancer induced cachexia which is already present at early stages. For drawing a general conclusion, this group (stage I/II) was too small in sample size. On the other hand, when the differing inflammatory statuses were considered, obvious differences were observed between the cancer patients for serum levels of TTR and RBP. Using 40 mg/l as cut-off for C-reactive protein (CRP) TTR and RBP serum levels were reduced in those individuals with increased CRP values. This clearly supports observations showing that the inflammation status greatly reduces TTR and RBP serum levels as a consequence of a reduced synthesis of this negative acute-phase protein in the liver [5].

Using immunological procedures we were recently able to show the presence of TTR in ascitic fluid from women with ovarian cancer, however no quantitative data, especially with regard to cancer stages, is as yet available [32]. In accordance to our previous semi-quantitative study, TTR in ascitic fluid was more than 100-fold lower when compared to its serum levels. This ratio is much lower in comparison to the one observed for RBP (Tab. 1). Based on the difference between their molecular masses, 55 kDa for the hetero-tetramer TTR and 21 kDa for RBP, one would expect a different ascites/serum ratio, as an inverse correlation exists between ascites/serum ratio and the mean of the molecular weight of various proteins [5]. From this observation one could assume that RBP and TTR are not transferred individually but rather as the complex usually present in serum [33]. In general, results support the hypothesis that the concentration of TTR and RBP in ascitic fluid is the result of a passive transfer from serum into the ascitic fluid. The accumulation of these and other serum constituents is mainly attributed to the increased capillary permeability caused by an increase in permeability-inducing factors such as the vascular endothelial growth factor (VEGF) [25].

These observations and the fact that no obvious differences in microheterogeneity between TTR from serum and ascitic fluid can be observed, both with regard to the known modification at the Cys^10 ^and the recently described truncated form, it can be assumed that all TTR in ascitic fluid originates through a passive transfer from serum. This is further supported by the observation that the tumor site itself does not express any immunoreactive TTR. It can not be excluded however, that the tumor site or components in the ascitic fluid may have proteolytic properties that possibly result in the unobserved modifications of TTR or other proteins.

With regard to the microheterogeneity of TTR in serum and ascitic fluid, the results support and confirm previous studies undertaken by us and others with regard to molecular variants of TTR in serum [17,34-36]. As in these studies, TTR in serum and ascitic fluid was dominant in four variants. The 118 Da larger variant is the S-cysteinylated form of the native TTR whilst the signal at 14,062 Da can be attributed to the S-glutathionylated TTR form [30,34,37]. As TTR contains only one cysteine residue (Cys^10^), the adduct must result when the Cys^10 ^residue forms a mixed disulfide with the amino acid cysteine, the dipeptide cysteinylglycine or the tripeptid glutathione. The shift in the mass spectrum of TTR variants towards the native TTR molecule mass in serum and ascites fluid after treatment with DTT indicates that the adducts are formed via the disulfide linkage at Cys^10^. In addition to this, we confirmed in serum of healthy and affected women as well as in ascitic fluid the presence of a smaller immunoreactive form of TTR with a molecular mass of 12,830 Da, which was recently identified as a truncated form of TTR lacking the NH_2_-terminal 10 amino acids [21]. Its presence in both serum and ascitic fluid supports once again the idea of passive transfer from serum into ascitic fluid during its accumulation.

## Conclusion

Results show that, although the microheterogeneity of TTR itself and the occurrence of possible immunoreactive fragments thereof in serum and ascites fluid is unaffected by the cancer. Absolute levels of TTR as well as RBP in serum are negatively affected by the disease and by inflammatory processes associated with the cancer. It cannot be excluded that other metabolic effects yet to be defined might interact with the cancerous process. Thus, to fully validate the specificity of TTR or any of its fragments as a biomarker for ovarian cancer, a careful selection of controls has to be implemented, including consideration of nutritional status and the presence of inflammatory processes especially the possible influence of various hepatic diseases.

## List of abbreviations used

BSA (bovine serum albumin)

CA125 (cancer associated antigen 125)

CRP (C-reactive protein)

Cys ^10 ^(solt cysteine residue on position 10 of each TTR subunit)

Da (Dalton)

DTT (dithioretiol)

EAM (energy-absorbing molecule)

ELISA (enzyme-linked immunoassay)

FIGO (International Federation of Gynecology and Obstertrics)

MALDI (matrix assisted laser desorption and ionization – time-of-flight – mass spectrometry)

MW (molecular weight)

RBP (retinol-binding protein)

SD (standard deviation)

TBS (Tris-buffered saline)

TOC (Tumor Bank Ovarian Cancer)

TTR (transthyretin)

## Competing interests

The author(s) declare that they have no competing interests.

## Authors' contributions

BG, JR, JS and FJS participated in the conception, design, data analysis and the writing of the manuscript. SH participated in the analysis and interpretation of MS. DK and AM participated in sample validation and data analysis. All authors have read and approved the last version of the manuscript.

## Pre-publication history

The pre-publication history for this paper can be accessed here:



## Figures and Tables

**Table 1:** Comparison of levels (mean ± SD) of TTR and RBP in serum and asciticfluid in relation to FIGO-stages and levels of CRP in ovarian cancer patients

	**serum (μg/mL)**		**ascitic fluid (μg/mL)**		**percentage of serum in ascitic fluid**	
	**TTR**	**RBP**	**TTR**	**RBP**	**TTR**	**RBP**

**Healthy control **n = 20	363.3 ± 105.5	55.8 ± 9.3	-	-	-	-
**All Stages ovarian cancer**	148.5 ± 96.7	22.5 ± 14.8	1.022 ± 0.239	4.632 ± 1.572	1.0 ± 0.6	26.2 ± 14.9
**Stage I/II ovarian cancer**	162.5 ± 69.4	34.2 ± 22.3	0.966 ± 0.375	3.580 ± 0.849	0.8 ± 0.6	14.5 ± 9.8
**Stage III ovarian cancer**	155.6 ± 107.6	19.5 ± 7.5	1.074 ± 0.185	4.800 ± 1.711	1.0 ± 0.6	27.2 ± 11.8
**Stage IV ovarian cancer**	129.0 ± 96.0	17.4 ± 7.3	0.965 ± 0.241	4.627 ± 1.672	1.2 ± 0.7	32.1 ± 23.3
**Ovarian cancer CRP > 40 mg/l**	116.0 ± 94.3	14.2 ± 4.3	1.047 ± 0.213	4.736 ± 1.886	1.4 ± 0.7	35.4 ± 17.2
**Ovarian cancer CRP < 40 mg/l**	166.3 ± 92.1	24.1 ± 12.5	1.005 ± 0.260	4.503 ± 1.415	0.8 ± 0.5	22.3 ± 11.1

All values for ascitic fluid (if available) are significantly different (p < 0.01) from serum value.

**Table 2:** Molecular mass of immunoprecipitated TTR (Da, mean ± SD) assigned to different forms^1 ^of TTR between serum and ascitic fluid of 20 representative women with ovarian cancer. Values in brackets represent mass differences of modified TTR in relationship to the native form of TTR (Da, mean ± SD).

	**TTR**	**cysTTR**	**cysglycTTR**	**glutTTR**
**serum**	13757.7 ± 7.1	13875.8 ± 12.8 (118.1 ± 13.7)	13923.6 ± 21.0 (166.8 ± 20.9)	14062.1 ± 24.7 (306.4 ± 23.9)
**ascitic fluid**	13752.7 ± 16.5	13876.9 ± 13.3 (124.2 ± 13.7)	13926.8 ± 11.1 (176.8 ± 1.8)	14042.5 ± 27.0 (294.6 ± 22.7)

^1 ^TTR = native TTR; cysTTR = TTR + cysteinylation; cysglycTTR = TTR + cysteinylglycine; glutTTR = TTR + glutathione; no significant differences between molecular masses of serum and ascitic fluid could be observed
